# Using chanarin-dorfman syndrome patient fibroblasts to explore disease mechanisms and new treatment avenues

**DOI:** 10.1186/s13023-025-03711-6

**Published:** 2025-04-24

**Authors:** Mor Angel, Yuval Kleinberg, Tanmoy Newaz, Victoria Li, Rinat Zaid, Keren Oved, Orly Dorot, Edward Pichinuk, Emily Avitan-Hersh, Ann Saada, Karin Weiss, Vanina Zaremberg, Galit Tal, Einat Zalckvar

**Affiliations:** 1 Department of Molecular Genetics, Weizmann Institute of Science, Rehovot, 7610001 Israel; 2 Blavatnik Center for Drug Discovery, Tel Aviv University, Tel Aviv, 6997801 Israel; 3 Department of Biological Sciences, University of Calgary, Calgary, AB T2N 1N4 Canada; 4 The Genetics Institute, Rambam Health Care Campus, Haifa, Israel; 5 Clinical Research Institute at Rambam (CRIR), Rambam Health Care Campus, Haifa, Israel; 6 Department of Dermatology, Rambam Health Care Campus, Haifa, Israel; 7 Metabolic Unit, Ruth Rappaport Children’s Hospital, Rambam Health Care Campus, PO Box 9602, Haifa, 3109601 Israel; 8 Rappaport Faculty of Medicine, Technion-Israel Institute of Technology, Haifa, 3109601 Israel; 9 Department of Genetics, Hadassah Medical Center, Jerusalem, Israel; 10 Faculty of Medicine, Hebrew University of Jerusalem, Jerusalem, Israel; 11 Department of Medical Laboratory Sciences Jerusalem Multidisciplinary College, Jerusalem, Israel; 12 The Mina and Everard Goodman Faculty of Life Sciences, Bar-Ilan University, Ramat- Gan, 52900 Israel

**Keywords:** Chanarin-Dorfman syndrome, Neutral lipid storage, Lipid droplets, Peroxisomes, Mitochondria, Drug repurposing

## Abstract

**Background:**

Chanarin-Dorfman syndrome (CDS) is a multisystemic autosomal recessive rare disorder. CDS is caused by variants in the abhydrolase domain containing 5 (ABHD5) encoding gene (*CGI-58*), which ultimately leads to excessive lipid storage, and therefore a high abundance of cellular lipid droplets (LDs). Although the molecular etiology of the disease was described many years ago, no treatment for CDS is currently available.

**Results:**

To further characterize the molecular basis of the disease and to uncover new treatment avenues, we used skin fibroblasts originating from a young patient diagnosed with CDS due to a homozygous nonsense mutation. We show that dysfunctional ABHD5 does not only affect LDs, but also influences other metabolic-related organelles; the mitochondria and peroxisomes. Additionally, we found that expressing functional ABHD5 in CDS patient cells reduced LD number. Finally, we developed and applied a high content-based drug repurposing screen based on a collection of ∼2500 FDA approved compounds, yielding several compounds that affected LD total area and size.

**Conclusions:**

Our findings enhance the understanding of the dysfunction underlying CDS and propose new avenues for the treatment of CDS patients.

**Supplementary Information:**

The online version contains supplementary material available at 10.1186/s13023-025-03711-6.

## Background

Chanarin-Dorfman syndrome (CDS) is a rare, autosomal recessive, neutral lipid storage disease (NLSD) associated with ichthyosis [1, 2]. The syndrome is typically characterized by the accumulation of lipid vacuoles (i.e., Jordans’ bodies) in neutrophils and is associated with the buildup of triglycerides (TGs) in various tissues and organs (e.g., muscle fibers, liver, skin) [3–6]. The excess in TGs can lead to a variety of clinical symptoms, such as fatty liver, hearing loss, ocular symptoms, and central nervous system involvement [4, 7, 8].

CDS is caused by bi-allelic pathogenic variants in the comparative gene identification (*CGI-58*) gene, located on chromosome 3p21. CGI-58 encodes the abhydrolase domain containing 5 (ABHD5) protein, a member of the α/β hydrolase protein family that plays an important role in the activation of the adipose triglyceride lipase (ATGL/PNPLA2) [8, 9]. ATGL is the rate-limiting enzyme in the release of fatty acids from TG stores, also termed lipid droplets (LDs). Therefore, loss-of-function mutations in the ABHD5 encoding gene reduces ATGL activation, which, in turn, leads to an accumulation of TGs and an increase in LD abundance in CDS patient cells [10].

Although CDS was first characterized almost half a century ago, currently there is no treatment for the disease [11]. Therefore, we aim to open the horizon to new treatment avenues for CDS; to pursue this, we used primary skin fibroblasts originating from a patient who was diagnosed with CDS due to a homozygous nonsense variant in ABHD5. We show that overexpression of the functional ABHD5 in these fibroblasts dramatically reduces LD number and that mitochondria and peroxisomes are affected in the CDS patient cells in comparison to control skin fibroblasts. Finally, to discover potential therapeutic compounds for CDS we developed and performed a high content-based drug repurposing screen, thereby identifying several compounds that decreased total LD area. In light of our finding, we wish to set the basis for the development of potential compounds and/or treatment strategies for CDS patients.

## Methods

### Subject

The patient is under the care of The Metabolic Clinic at Ruth Rappaport Children’s Hospital. The study was approved by the Institutional Review Board of Rambam Medical Center (0038 − 14) and the parents consented to the performance of skin biopsies, functional studies and publication of the results.

### Sanger sequencing

Sanger sequencing was performed according to standard methods on DNA samples of the proband and parents. Primers used for the *ABHD5 (CGI-58)* c.700 C > T variant on exon 5: Forward: GTACGAGCTACTCGCCACG Reverse: TGATACAAATCTTCTGGACCACTG.

### Cell culture

Human HeLa S3 cells (a gift from Orly Laufman, Weizmann Institute of Science) were maintained in DMEM/F-12 medium containing glutamine (Gibco, USA) supplemented with 10% fetal bovine serum (FBS, Gibco, USA) and 1% Penicillin-Streptomycin-Neomycin (Biological Industries, Israel). Primary skin fibroblasts from the patient (SK-44) were grown in DMEM (Gibco, USA) filtered medium, supplemented with 10% FBS (Gibco, USA) and 1% Penicillin-Streptomycin-Neomycin (Biological Industries, Israel). GM00498 cells (Coriell Institute, USA) served as control skin fibroblasts cells, matched by age and gender to the patient characteristics, and were grown in MEM medium (Biological Industries, Israel) containing non-essential amino acids (NEA, Biological Industries, Israel) and 1% L-Glutamine, supplemented with 15% FBS (Gibco, USA) and 1% Penicillin-Streptomycin-Neomycin (Biological Industries, Israel). For mitochondria and peroxisome imaging and analysis, all cells were grown in DMEM supplemented with 10% fetal bovine serum (FBS, Gibco, USA) and 1% Penicillin-Streptomycin-Neomycin.

For testing the effect of the hits identified in the drug repurposing screen, cells were treated with Lomitapide (10µM, SML1385, Sigma), Benzalkonium Chloride (10µM, 12060, Sigma), Tafenoquine (10µM, SML0396, Sigma) or Mitoquinone (10µM, ab285406, Abcam), all dissolved in DMSO. Control fibroblasts FSE-hTERT (immortalized fibroblasts from a male baby foreskin) and SK-44-hTERT (immortalized CDS patient fibroblasts) were grown under similar conditions as patient fibroblasts.

### Plasmids and transfections

Wild type and mutant (C700T) *ABHD5* coding DNA were synthesized by GenScript (USA Inc.) and cloned directionally in pcDNA3.1(+)-N-eGFP using KpnI and BamHI restriction enzyme sites. HeLa S3 cells were transfected with plasmids expressing the GFP-ABHD5-mutant or GFP-ABHD5-WT plasmids (1 µg/ml) for 24 h using Lipofectamine 2000 (Invitrogen, USA). Patient immortalized cells (SK-44-hTERT) were electroporated with GFP-ABHD5-WT using the DT-130 program in the 4D-nucleofactor (Lonza, Switzerland). 2 µg plasmid was electroporated per 600,000 cells in P3 solution (Lonza, Switzerland).

### Immunofluorescence

Cells were grown on coverslips 18-mm (Bar-Naor, Israel) in a 12 well plate and fixed in 4% paraformaldehyde (PFA) (Electron Microscopy Sciences, USA) in PBS (Sigma, USA) for 20 min. Cells were permeabilized in 0.01% Digitonin (Sigma, USA) solution in DPBS (without calcium and magnesium, Sigma, USA) for 30 min, and blocking was applied using 5% BSA- bovine albumin fraction V (MP Biomedicals). Then, cells were incubated with primary antibodies for 1 h, washed with 1X DPBS, and incubated with secondary fluorescent antibodies. The nucleus was stained with Hoechst H33342 (1:1000, Sigma, USA) and coverslips were mounted in Immu-Mount mounting medium (Thermofisher scientific, USA). Primary antibodies used were: anti-PEX14 (1:100, Ptglab, 10594-1-AP), anti-TOM20 (1:150, Santa Cruz, SC-17764). Secondary antibodies were purchased from Invitrogen: AlexaFlour 568 anti-rabbit (1:500, A11036), AlexaFlour 568 anti-mouse (1:500, A11031), AlexaFlour 488 anti-mouse (1:1000, A11029), AlexaFlour 488 anti-rabbit (1:1000, A11034) and AlexaFlour 647 anti-rabbit (1:1000, A-21244). Lipid droplets were stained using BODIPY 493/503 (1:1000, Invitrogen, D3922), or BODIPY 558/568 (1:2000, Invitrogen, D3835) applied with the secondary antibody.

### Imaging

Images were acquired with an Andor Dragonfly 505 confocal spinning disk system, operated via Fusion software. The system is equipped with a Leica Dmi8 inverted microscope, and images were collected using a Plan Apo 63 × (1.40 N.A.) oil immersion lens. All images were edited using Fiji [12].

### Western blotting

Cells were washed with cold 1XDPBS, and proteins were extracted using RIPA lysis buffer (Sigma, USA) containing EDTA-free protease inhibitor cocktail (Sigma, USA). The samples were then placed on ice for 20 min, and were centrifuged at 14,000 g for 20 min. Protein samples were separated by SDS-PAGE using a 4–20% gradient gel (Bio-Rad) and then transferred onto 0.45-µm nitrocellulose membrane (Pall Corporation) using the Trans-Blot Turbo transfer system (Bio-Rad). Membranes were blocked in SEA BLOCK buffer (Thermo Scientific; diluted 1:5 in DPBS) for 1 h at RT and subsequently incubated overnight at 4 °C with primary antibodies diluted in a 2% wt/vol BSA/DPBS solution containing 0.01% NaN3. Primary antibodies used were: anti-ABHD5 (1:250, Sigma, HPA035851), anti-ATGL (1:1000, Cell signaling, 2138 S) and anti-Vinculin (1:1000, Abcam, ab129002). After washing, membranes were probed with secondary antibodies purchased from Abcam (800CW Goat anti-Rabbit IgG, ab216773 or Goat anti-Rabbit IgG H&L 680, ab216777) diluted 1:10,000 in 5% wt/vol nonfat milk/Tris-buffered saline with 0.05% Tween 20 (TBST) for 1 h at RT. Blots were washed and imaged on the LI-COR Odyssey Infrared Scanner.

### ATP and COX assay

Evaluation of ATP production in microtiter wells was carried out essentially as we have previously described [13]. Briefly, cells were maintained in permissive glucose containing (GLU) DMEM-high glucose medium supplemented with 15% fetal calf serum (FBS, Gibco, USA), 50 µg/ml uridine (Sigma, USA), 110 µg/ml pyruvate and 2mM glutamine at 37 °C in 5% CO_2_. 3 × 10^3^ cells per well were seeded on two parallel 96-well cell culture plates and grown for 24 h at 37 °C, 5% CO_2_. The following day the medium was replaced with fresh GLU medium, containing 5% FBS and 2mM glutamine, and growth continued for another 72 h. Cell content was measured by spectrophotometry after glutaraldehyde fixation and methylene blue (MB) stain. Mitochondrial ATP production was measured in digitonin-permeabilized cells after incubation with glutamate and malate (Sigma, USA) in the presence of ADP and analyzed by luciferin–luciferase using the ATPlite Luminescence Assay System (PerkinElmer Life Sciences) and normalized to MB. Luminometric and fluorometric measurements were performed on a BioTek Synergy HT microplate reader (AgilentTechnologies, Wokingham UK).

For COX assay, fibroblasts were washed in PBS, detached by scraping, centrifuged; the dry pellet was stored at -80 C until assay. Enzymatic assays were performed as previously described [14]. Complex IV (cytochrome c oxidase) was measured by following the oxidation of reduced cytochrome c at 550 nm.

### Drug repurposing screen and statistical analysis and quantifications

1500 patient skin primary cells or GM05400/GM05381 control cells (skin fibroblasts, NIGMS Human Genetic Cell Repository) were seeded per well, on 384 well plates, and grown in RPMI medium (Gibco, supplemented with 10% FBS, 1% Penicillin-Streptomycin-Neomycin (PSN), 1% L-Glutamine, 1% sodium pyruvate (SP), 1% non-essential amino acids (NEA). The next day, the medium was replaced, and cells were treated with compounds at 10 µM concentration with a final DMSO concentration of 0.1% for 24 h. Patient skin primary cells with 0.1% DMSO were served as negative control (12 wells per plate). GM05400/GM05381 cells with 0.1% DMSO were served as positive controls (12 wells per each cell type per well). The drug library-Drug Repurposing Hub (Broad Institute)– an annotated collection of FDA-approved drugs, contained 2468 drugs. 30 min prior to imaging, cells were stained using BODIPY 493/503 (10µM, 8160 Setareh Biotech) to mark lipid droplets, Calcein Red (0.3 µM, C34851 Thermo Scientific) for cell segmentation, and Hoechst (1.1 µg/ml, H1399 Thermo Scientific) for nuclear staining. Cells were imaged using IN Cell Analyzer 2200 (Cytiva), lens 20X/0.75, and 12 fields were taken per well. Four parameters were measured relying on the BODIPY signal- LD area, LD Compactness, LD total area, and LD form factor in control and patient fibroblasts. Then, for every parameter a separate scatter plot was generated, and for each compound the distance in standard deviation from the median of the samples (SSMD) was calculated. Significancy was determined by exceeding 2SD threshold (area, compactness and total area), or 4SD threshold (form factor). The final hits were chosen by showing significancy in all four parameters individually. For mitochondrial characterization, cells were stained with TMRE (0.05 µM, T-669 Thermo Scientific) and MitoTracker Deep Red (0.06 µM, M22426 Thermo Scientific) for 30 min prior to imaging. Image analysis was done using IN Cell 2200 image analysis software, for each compound concentration, we used four wells, and 12 fields were taken per well. Statistical analysis was carried out using two-tailed T-tests, or by Mann-Whitney-U-test. All graphs were plotted using Python.

Quantifications for peroxisome number and size were carried out using Fiji, and statistical analysis and graphs were plotted using Prism GraphPad (version 10). Cells were counted manually using Hoechst DNA staining (Thermo Scientific). Then, peroxisomes number and size were quantified using Fiji by particle segmenting of peroxisomes in the cytoplasm and setting a threshold. Size and quantity of peroxisomes (size and area of particles) were calculated by Fiji. Significancy was determined using two-tailed T-test or using two-tailed Mann-Whitney test in case that data did not meet normal distribution.

**Fig. 1:**
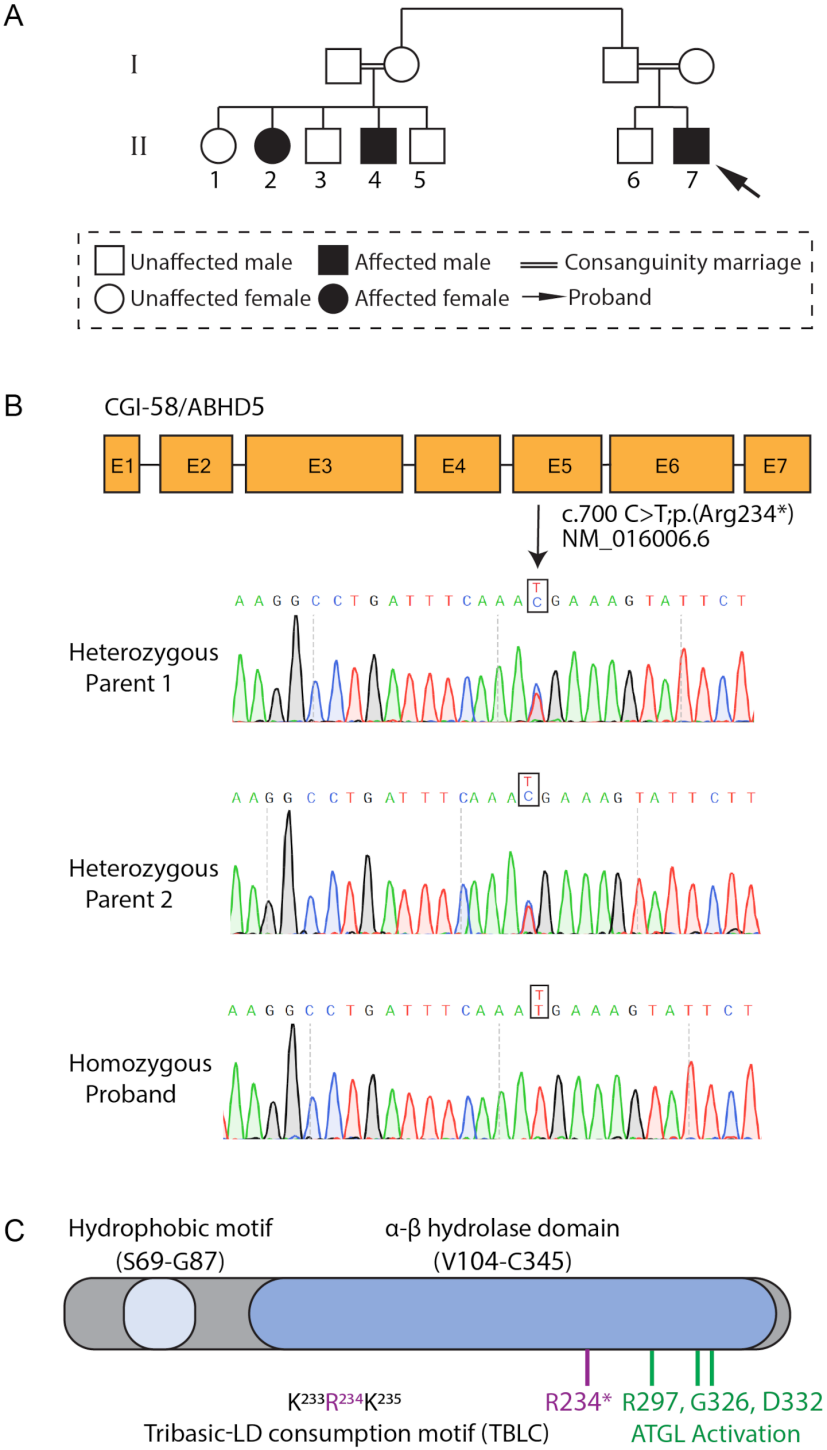
Characterization and diagnosis of a CDS patient. (**A**) Patient pedigree. The diagnosed patient (i.e., proband) is marked with an arrow. (**B**) Illustration of the CGI-58 gene, encoding for the ABHD5 protein, as sequenced by Sanger sequencing. Exons are marked with E. Arrow marks the location of the proband mutation. (**C**) Domain organization of the ABHD5 protein. The human ABHD5 contains 349 amino acids, comprising a hydrophobic domain (LD-binding site) and an α/β hydrolase domain; the three residues involved in ATGL activation (R297, G326 and D332 are highlighted in green). The R234* ABHD5 mutation reported here (red) is localized to the tribasic-LD consumption motif (TBLC)

All experiments were performed in at least three biological replicates (experiments), and the results consisted across these replicates.

Statistical analysis of ATP production assay and COX assay was carried out using an unpaired two tailed t-test with GraphPad Prism (version 10), *p* < 0.05 was considered statistically significant.

### Human ABHD5 and ATGL complex modelling

The human ABHD5 and ATGL heterodimer complex structure was predicted by AlphaFold-Multimer [15, 16] in the molecular visualization program UCSF ChimeraX (developed by Resource for Biocomputing, Visualization, and Informatics at the University of California, San Francisco) [17]. The input for the predictions were the amino acid sequence of human ATGL, and either the full-length amino acid sequence of ABHD5 or the truncated mutant lacking 116 residues from the carboxy-end. The matchmaker function within UCSF ChimeraX was used to superimpose predicted structures. Calculations were done with AlphaFold v2.3.2 and local ColabFold v.1.5.4. The PyMOL Molecular Graphics System, Version 2.5 (Schrödinger, LLC.) was used for coloring and molecular visualization of specific regions in the protein complex.

## Results

### A patient diagnosed with Chanarin-Dorfman syndrome caused by a nonsense mutation

A patient was referred to the metabolic clinic at Rambam hospital in 2019 at 4 months of age due to ichthyosis. He is the second child of consanguineous Arab Muslim parents with a known family history of CDS (Fig. 1A), and thus diagnosis of CDS was suspected. Currently 5 years old, the patient has general good health, normal growth, and normal development. He does not have hepatomegaly, however on ultrasound there is increased echogenicity of the liver, suggestive of liver steatosis. He has elevated transaminases (alanine aminotransferase 93 U/L, normal range 0–55; aspartate aminotransferase 98 U/L, normal range 5–34), with normal gamma-glutamyl transferase, albumin, bilirubin and lipid profile. Since 19 months of age, he has been treated with a low-fat diet supplemented with medium-chain triglycerides [18].

**Fig. 2:**
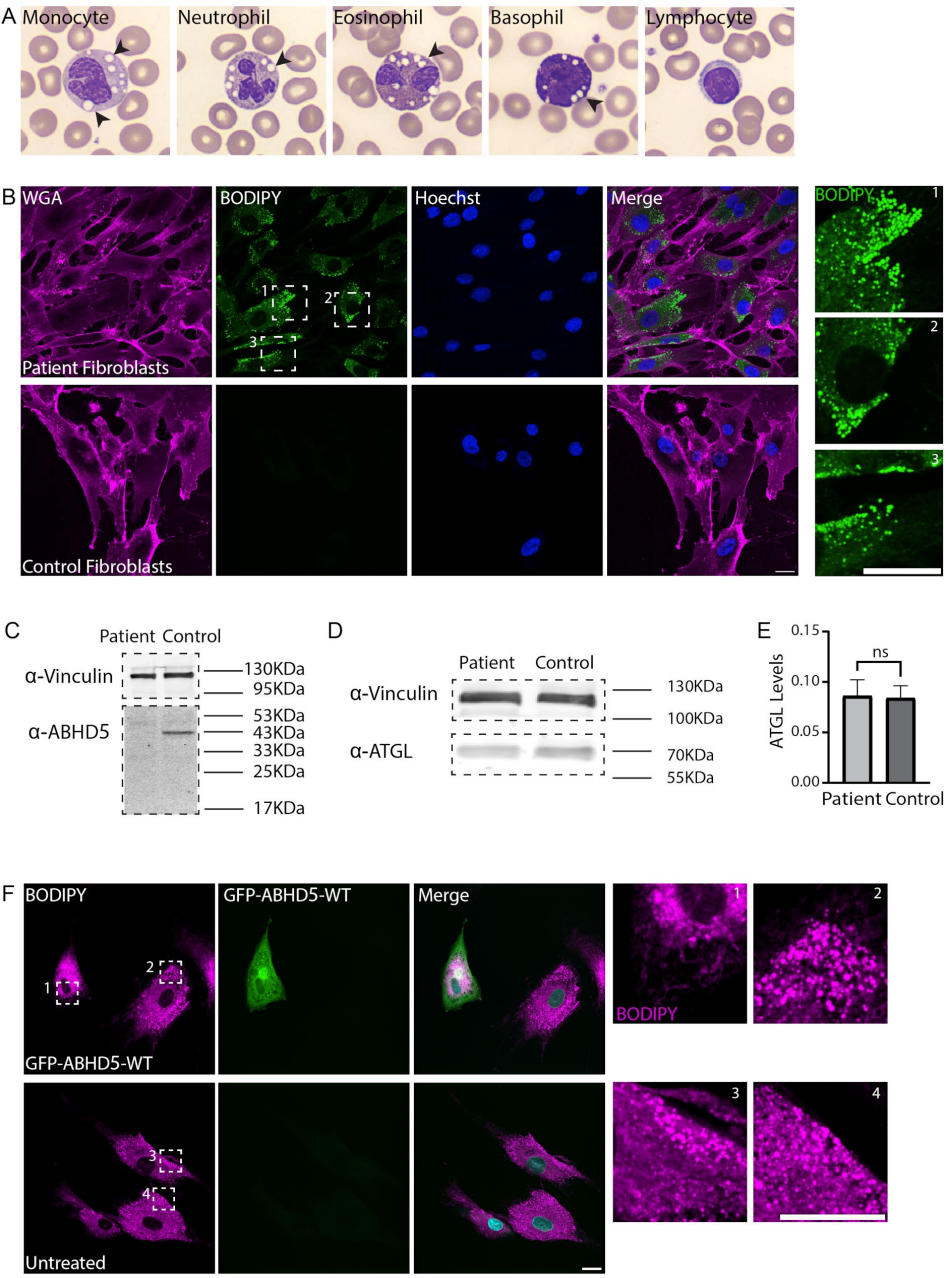
LDs accumulate in the patient fibroblasts and can be reversed by overexpressing ABHD5-WT. (**A**) Images of representative cells detected in the CDS patient. Lipid vacuoles are marked by black arrowheads. (**B**) Primary skin fibroblasts derived from the proband and from a healthy individual (GM00498 cells) were stained with WGA for membrane segmentation (magenta), and with BODIPY to mark LDs (green). DNA was stained with Hoechst H33342 (blue). Enlargements of designated areas are in the boxed regions at right as numbered. Scale bars = 20 μm. (**C-D**) Western blot analysis of proteins extracts from skin fibroblasts derived from CDS patient cells, and from control skin fibroblasts cells (GM40098). Anti-Vinculin was used for loading control. Blots were incubated with (**C**) anti-ABHD5 and (**D**) anti-ATGL. (**E**) Quantifications of ATGL signal levels as seen in D. Data were analyzed using Fiji, and statistical analysis was carried out by two-tailed T-test (*n* = 3, ns = non-significant). Bar graph illustrates the mean ± SD. (**F**) Immortalized skin fibroblasts originating from the proband were electroporated with GFP-ABHDT-WT plasmid (green), followed by fixation after 24 h, and BODIPY staining to mark LDs (magenta). The experiment was performed three times (*n* = 12 positive cells). Enlargements of designated areas are in the boxed regions at right as numbered. Scale bar = 20 μm

Due to family history, and to verify that the genetic cause of the disease stems from a mutation in the ABHD5-encoding gene, *ABHD5* Sanger sequencing was performed and revealed a homozygous nonsense variant c.700 C > T, p.Arg234* in exon 5 out of 7 (Fig. 1B). Additional cases with the same variant have been reported in the past [19] (Supplementary Fig. 1). This mutation produces a premature stop codon at position 234 of the tribasic-LD consumption (TBLC) motif (K233 R234 K235) [20] within the ABHD5 protein. The C-terminal portion of the protein derived from exons 5–7, including amino acids R297,G326 and D332, which are crucial for the activation of ATGL, the rate limiting lipase in TG degradation, are completely lacking in the truncated ABHD5 [20, 21] (Fig. 1C). Since currently no treatment is available for CDS patients, we decided to further study the molecular mechanism of the disease, focusing on this ABHD5 mutant.

### ABHD5 is not detected in CDS patient cells, while supplementing functional ABHD5 to patient fibroblasts decreases LD abundance

The diagnosis of CDS patients relies on the presence of clinical symptoms such as ichthyosis, followed by the detection of LDs in peripheral blood smear cells (Jordans’ anomaly) [4]. Indeed, vacuoles were observed in cells of myeloid origin (i.e., monocytes, neutrophils, eosinophils, and basophils), but not in cells of lymphoid origin (i.e., lymphocytes), suggesting that LDs are found in the patient myeloid cells (Fig. 2A). Furthermore, in line with previous observations [22, 23], LDs accumulated in patient fibroblasts and not in the control fibroblasts (Fig. 2B) implying that patient fibroblasts are a good candidate model to study the molecular mechanism of the disorder.

**Fig. 3:**
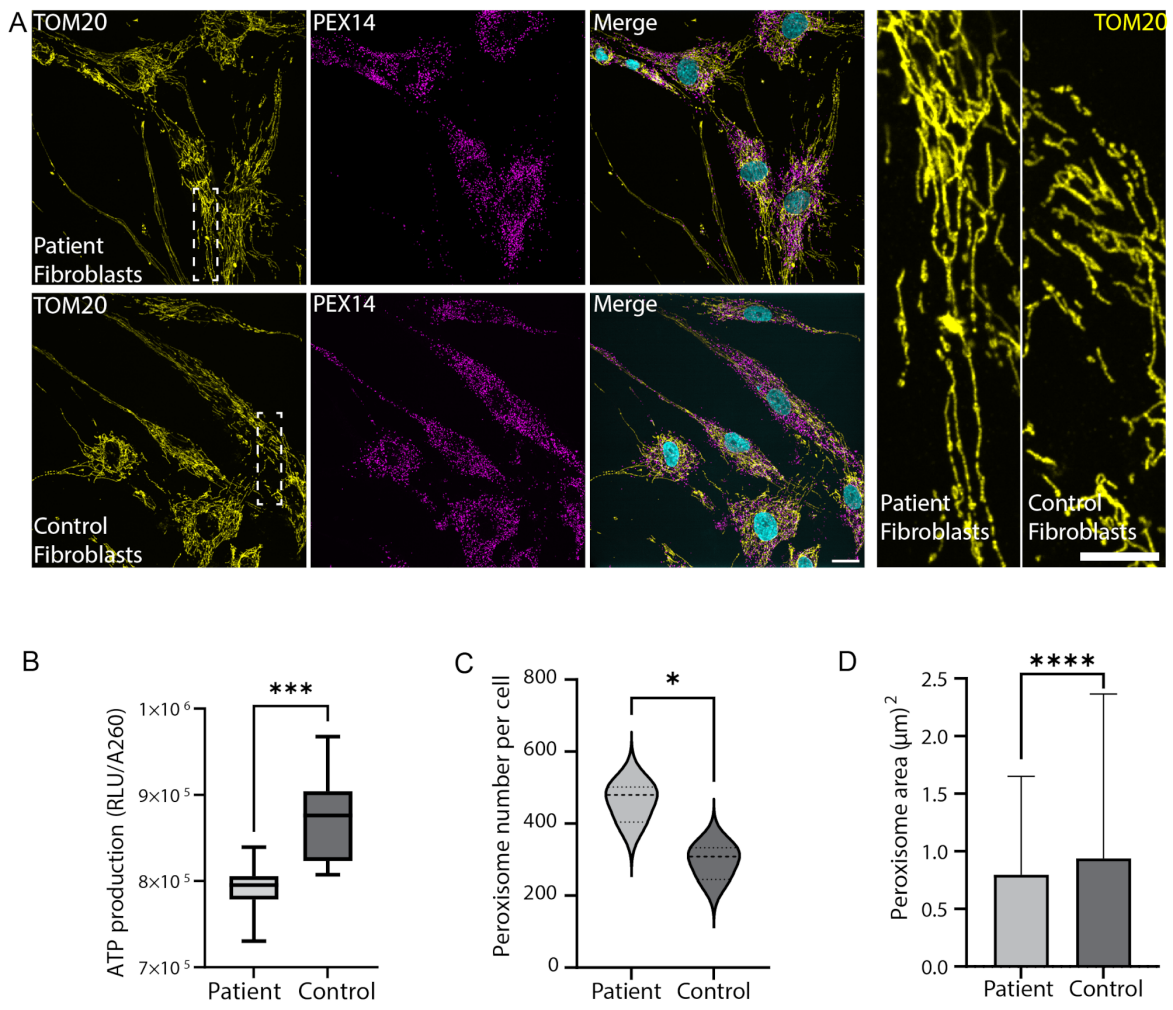
Mitochondria and peroxisomes are affected in CDS patient fibroblasts. (**A**) Primary skin fibroblasts originating from the proband, and control skin fibroblasts (GM00498) were immuno-stained with anti-TOM20 to mark mitochondria (yellow), and with anti-PEX14 to mark peroxisomes (magenta). DNA staining is in cyan. Scale bar = 20 μm. Enlargements of designated areas are in the boxed regions at right. Scale bar = 10 μm. (**B**) Box plot represents ATP production in the patient and control (GM00498) skin fibroblasts expressed as relative fluorescence units (RFU) per cell content measured by methylene blue absorbance 620 nm (A620) (***p < 0.001, *n* = 10), (**C**) Violin plot quantifying number of peroxisomes per cell as seen in A. Data were analyzed using two-tailed T-test (*p < 0.05, more than 100 cells were quantified per patient or control cells, *n* = 3 independent biological replicates). (**D**) Bar graph quantifying area of peroxisomes as seen in A. Data were analyzed using two-tailed Mann-Whitney test (****p < 0.0001, *n* > 33,000 peroxisomes per patient or control cells, more than 150 cells were quantified). Bar graph illustrates the mean ± SD

Patient skin fibroblasts are commonly used to study the molecular mechanism underlying rare genetic disorders. Hence, to examine if the truncated ABHD5 protein is present in the patient cells, a skin biopsy taken from the patient was used to perform western blot analysis of the patient and control fibroblast cell lysates using the anti-ABHD5 antibody that recognizes the N’ of ABHD5. Interestingly, while ABHD5 could be detected in the control cells, we could not detect the mutant protein in the patient cells (Fig. 2C). This suggests the mutant protein is not stable and prone to degradation, or alternatively, that is not expressed due to nonsense mediated mRNA decay. Since it is known that ABHD5 plays an important role in ATGL activation leading to lipolysis [21], we were curious to examine if ATGL levels are affected in the patient cells. Interestingly, we found that ATGL levels were similar in patient and control cells (Fig. 2D, E).

Following our finding that ABHD5 was not detected in the patient cells, we asked whether a treatment strategy that relies on the stabilization of the mRNA transcript and/or the protein is suitable. For that aim, we cloned a plasmid containing the mutated c.700 C > T ABHD5 tagged on the amino terminus (N’) with GFP and transfected it into HeLa S3 cells, which typically show LDs [24] and are often used to study lipid consumption (i.e., degradation of triacylglycerols). Interestingly, the GFP-ABHD5-mutant protein was detected (Supplementary Figure S2A), but did not localize to LDs, in contrast to wild type GFP-ABHD5 (Supplementary Figure S2B). Importantly, these results suggest that first, the GFP-ABHD5-mutant protein is stable, and second, reassure a validation that the anti-ABHD5 antibody that was used on patient cells lysates (Fig. 2C), indeed, recognizes the N- terminal region of ABHD5. We then questioned if the stabilized ABHD5-mutant protein could bind ATGL. Based on the Sanger sequencing results (Fig. 1B), the mutant protein is expected to end at Lys233, thereby lacking 116 amino acids from the carboxyl-end. To better understand the potential impact of such truncation on the interaction between ABHD5 with ATGL, we modelled the interaction with AlphaFold and visualized it using ChimeraX (Supplementary Figure S2C-F). Interestingly, the carboxyl terminus (C’) of ABHD5 is predicted to interact with a grove of ATGL, where three amino acid residues that are critical for ATGL activation [21], R297, G326 and D332, are localized (Supplementary Figure S2C, D). Lack of the C’ in the mutant ABHD5 is predicted to weaken the interaction, inducing conformational changes in ATGL (Supplementary Figure S2E, F). This weak interaction between the mutant ABHD5 and ATGL may explain why the mutant ABHD5 does not localize to LDs in HeLa S3 cells. Altogether, these results suggest that stabilizing the mutant ABHD5 might not be a suitable treatment strategy for the patient.

Since we observed that the stable ABHD5-mutant is not localized to LDs, and is predicted to have a weaker interaction with ATGL, we wondered if the overexpression of ABHD5-WT, that can promote the consumption of LDs [20], could reduce LD abundance in the patient fibroblasts. Indeed, we found that overexpressing GFP-ABHD5-WT in immortalized patient cells reduced the LD abundance (Fig. 2F). This implies that supplementing a functional ABHD5 to the CDS patient cells can reduce LD accumulation.

### CDS alters peroxisomes and mitochondrial properties

In recent years, evidence has emerged that peroxisomes, mitochondria, and LDs communicate with each other to maintain cellular lipid homeostasis [25–28]. Since LDs accumulate in the patient fibroblasts, we were motivated to characterize whether changes in other metabolic-related organelles- mitochondria and/or peroxisomes- occur in these cells. Interestingly, mitochondria, identified by staining for TOM20, were hyperfused in patient fibroblasts compared to control cells (Fig. 3A). We then aimed to investigate if the observed mitochondrial changes in morphology would also affect its function. Indeed, a significant reduction in the patient cells ATP production was detected compared to control fibroblast cells (Fig. 3B). To further evaluate mitochondrial function, we measured the activity of cytochrome c oxidase (COX) complex (IV). Interestingly, we found that COX activity in the patient cells was lower compared to the control cells (78 and 161 nmol/min/mg, respectively). We then asked if the reduction in mitochondrial function at the cellular level was due to decreased mitochondrial content per cell, or due to less active mitochondria. Interestingly, staining of the fibroblasts with MitoTracker to measure total mitochondria, and with tetramethylrhodamine, ethyl ester (TMRE) to label active mitochondria, showed that although there are more mitochondria in the patient cells, they may be less functional in comparison to the control cells (Supplementary Figure S3A-C). In addition to mitochondrial alterations in patient cells, we also identified changes in peroxisomes; quantifications revealed that there are more and smaller peroxisomes (identified by staining for PEX14) in the patient cells compared to the control cells (Fig. 3C, D). Altogether, this implies that dysfunctional ABHD5 not only affects LDs, but also affects, directly or indirectly, other metabolic-related organelles such as mitochondria and peroxisomes.

### Drug repurposing screen reveals compounds affecting LD size and quantity in CDS fibroblasts

**Fig. 4:**
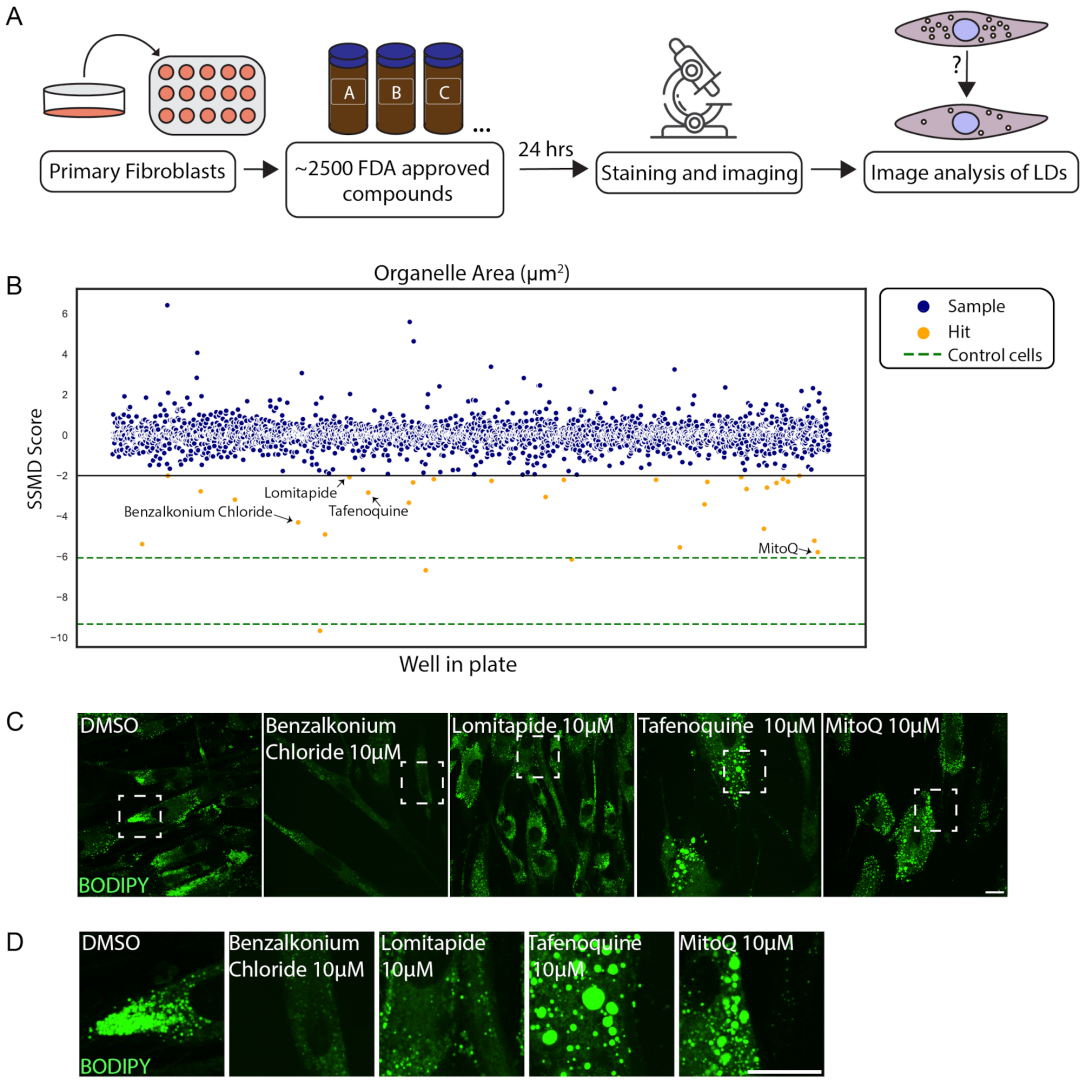
Drug repurposing screen on CDS patient cells reveals drugs that reduce LD total area in the patient fibroblasts (**A**) Illustration of high content drug repurposing screen. (**B**) Scatter plot representing all compounds screened and analyzed for total LD area. Compounds below − 2 (line marked in black) are determined as hits (yellow), that found significant in all four categories (i.e., compactness, form factor, area and total area). Other samples that were tested are marked in blue, and the score for control cells (GM05400) as a reference is marked in green. Black arrows point at the four compounds shown in C. (**C**) Primary skin fibroblasts obtained from the proband were treated with 10µM Benzalkonium Chloride, Lomitapide, Tafenoquine or MitoQ for 24 h. Cells were fixed and stained with BODIPY to mark LDs (green). (**D**) Enlargements of the marked boxes in C. Scale bar = 20 μm

Based on the LD accumulation in the CDS patient fibroblasts that can be easily detected by BODIPY staining (Fig. 2B, Supplementary Figure S3D), we established a high content-based screening system to identify compounds that reduce LD accumulation. We chose to perform a drug repurposing screen on the patient primary fibroblasts; we applied ∼2500 FDA approved compounds from the Drug Repurposing Hub (Broad Institute)–an annotated collection of FDA-approved drugs library, and looked for changes in LDs based on BODIPY intensity (Fig. 4A), focusing on four main parameters: compactness, form factor, LD area and LD total area, with an appropriate Z’ factor value (Supplementary Tables 1 and 2). The screen revealed several compounds (i.e., hits) that potentially affect total area, compactness, or LD area (Fig. 4B). We then determined significancy for each compound by calculating the distance in standard deviation from the median of the samples (SSMD). This was applied for the four parameters separately; we searched for compounds affecting all four parameters significantly and thus we chose to focus on four main hits - Mitoquinone (MitoQ), a synthetic analogue of coenzyme Q10 that serves as an anti-oxidant [29, 30], Lomitapide, a lipid-lowering drug used to treat familial hypercholesterolemia [31, 32], Tafenoquine, an anti-malaria drug [33] and Benzalkonium Chloride, a cationic surfactant that was used as a positive control to observe reduction in LDs as it was previously shown to alter lipid homeostasis [34, 35].

Reductions in measured total area of LDs may result from different changes in LDs, such as reduced LD number or size. To further validate the results obtained from the high content screen, and to better characterize the effect on LDs, we used fresh compounds and visualized the LDs by confocal microscope. Interestingly, while Benzalkonium Chloride and Lomitapide led to a decrease in LD quantity and size, MitoQ and Tafenoquine increased the size of the remaining LDs (Fig. 4C, D). Ultimately, we identified compounds that affected LD total area in CDS patient cells, with differing effects on LDs, suggesting that they may act through different mechanisms.

## Discussion

In this study, we used primary skin fibroblasts, derived from a CDS patient, who carries a nonsense mutation in ABHD5, to further characterize the molecular basis of the disorder and to establish a framework for identifying potential treatment avenues.

A simple way to diagnose CDS is by observing an abundance of LDs in cells. These LDs can be found in a variety of cells such as blood, skin, liver, and bone marrow [36–40]. Moreover, it was previously demonstrated that primary skin cells obtained from NLSD patients is an invaluable resource for studying the molecular base of pathology, and that LDs can be easily detected using microscopy-based methods in these cells [5]. Hence, we chose to use skin fibroblasts in our study.

Consumption of lipids that are stored in LDs (lipolysis) is mainly mediated by the triglyceride lipase- ATGL (PNPLA2), the hormone-sensitive lipase (HSL) and by monoacylglycerol lipase (MGL). Specifically, ATGL has been shown to be the rate limiting step in TG lipolysis and crucial for LD disassembly in adipocytes, and it also has an important role in LD degradation in other non-adipocytes cells [10, 41, 42]. ATGL levels are dynamic and can change under different metabolic and physiological conditions. For instance, a decrease in ATGL levels is observed in different types of cancer cells [43], which mostly rely on glycolysis and demonstrate hypoxia. Interestingly, we found that although the truncated ABHD5 is not detected in the patient cells, and the CDS skin fibroblasts contained a high quantity of LDs, ATGL levels were similar to control cells. Expressing the truncated ABHD5 protein in HeLa S3 cells did not result in its localization to LDs, and molecular modeling of ATGL in complex with truncated ABHD5 predicts a weaker interaction between the two proteins, suggesting a potential mechanism by which ATGL activation can be negatively affected. Yet, there is much more to discover, and other possible mechanisms should not be excluded. For example, it was shown that ABHD5 can also activate PNPLA1 [44], and that mutations in PNPLA1 lead to LD accumulation and ichthyosis [45], similar to mutations in ABHD5. Thus, it is possible that alterations in PNPLA1 levels or in its activation are more relevant in CDS fibroblasts. Strikingly, overexpressing ABHD5-WT in the patient cells reduced LDs. Recently, the FDA approved the first topical gene therapy for treatment of skin wounds of patients suffering from dystrophic epidermolysis bullosa (DEB) [46]. Previous studies reported that most of the ABHD5 characterized mutations in CDS patients lead to the generation of truncated proteins, while the others are missense mutations that could partially reduce the activity of ABHD5 [8, 47]. Hence, a potential avenue to treat the ichthyosis from which the CDS patients suffer is topical gene therapy of ABHD5.

LDs play an important role in cellular metabolism and energy homeostasis [48] and communicate with other metabolic organelles such as peroxisomes and mitochondria [49]. Here we showed that mitochondria are elongated with reduced membrane potential in the CDS patient fibroblasts compared to control fibroblasts. Furthermore, we show that although CDS is considered a LD-associated disease, there is a significant impact on mitochondrial function, including reduction in ATP production and COX activity. These findings are in line with additional studies that demonstrated that knocking out ABHD5 affected mitochondrial morphology and significantly reduced basal mitochondrial oxygen consumption rates and mitochondrial oxidative capacity [50, 51]. In addition, it was previously shown that starvation promotes the phosphorylation of the pro-fission dynamin related protein 1 (DRP1/DNM1L), which, in turn, leads to unrestricted mitochondrial fusion and increased activation of ATP synthase [52]. Interestingly, alternation in energy production was observed, due to a reduction of Krebs cycle substrates (i.e., mono and diglycerides) in NLSD-M (NLSD with myopathy) patients. Following these observations, a compound based on a triglyceride was tested on NLSD-M patient-derived fibroblasts, that ultimately led to a better mitochondrial respiration and an increased glycolysis [53].

Peroxisomes have a major role in energy and lipid homeostasis as well; they are essential for the breakdown of specific fatty acids that cannot be oxidized in the mitochondria [54]. Here, we found that the CDS patient cells display more peroxisomes, yet they are smaller compared to control cells. Notably, it was previously shown that a decreased oxidation of very long and long chain FAs is associated with ABHD5 perturbation [55]. Peroxisomes are also important for the synthesis of docosahexaenoic acid (DHA), which has been associated with an increased LD biogenesis [56, 57]. These are consistent with the observation that the number of peroxisomes was increased in the CDS patient cells, yet their size decreased in comparison to control fibroblasts. Interestingly, it was recently demonstrated that the peroxisomal targeting factor, PEX5, physically interacts with ATGL and is mediating lipolysis independently to ABHD5 [25]. It is possible that when ABHD5 is dysfunctional, ATGL is targeted to LDs, at least partially, by PEX5. This could potentially affect the availability of PEX5 to target proteins to the peroxisome matrix and lead to the presence of more and smaller peroxisomes. Interestingly, more and smaller peroxisomes were observed in yeast cells in the absence of Pex5 [58]. The alterations observed in mitochondria and peroxisomes imply that not only LDs, but also other metabolic organelles might be affected in CDS patients [59]. In the future, it would be valuable to investigate whether restoration of ABHD5-WT in the patient’s cells affects not only LDs, but also the morphology and/or function of mitochondria and peroxisomes. Additionally, further study of the molecular mechanisms by which dysfunctional ABHD5 impacts mitochondria and peroxisomes would be insightful.

Although CDS was first described in 1974 [60], currently no treatment is available for patients. Since LD accumulation can be easily visualized in patient fibroblasts, and since the skin is affected in the CDS patients who suffer from ichthyosis, we established and performed a drug repurposing screen in patient skin fibroblasts to find a potential therapeutic compound. Importantly, drug repurposing screens is a well-established, efficient, rapid and relatively cheap strategy that is now commonly used to identify potential treatments for various rare diseases [61]. After characterizing various parameters such as the effect of the compound on LD total area, we chose to focus on four main compounds. Both Tafenoquine, a drug that is used to treat malaria [33], and MitoQ, an antioxidant mimicking the mitochondrial antioxidant coenzyme Q10, which can decrease mitochondrial oxidative damage, led to a reduced total LD area but with enlargement of remaining LDs. Importantly, MitoQ was shown to reduce LDs in mice and rats fed with rich fatty acids diet and is already involved in several clinical studies for other diseases [30, 62, 63]. In contrast, Benzalkonium chloride, a quaternary ammonium salt that has anti-microbial properties, and Lomitapide, which is used to treat hypercholesterolemia, led to a decrease in both LD size and abundance. Moreover, it was shown that Lomitapide affects LD accumulation in Triple Negative Breast Cancer (TNBC) cells [32, 34]. Although we only used Benzalkonium chloride as a positive control and not as a potential treatment, it was previously shown that it alters lipid and cholesterol metabolism [35]. Interestingly, it was described that Benzalkonium chloride acts on cell membranes yet did not induce changes in neutral lipids and LD number in Human Corneal Epithelial (HCE) cells [64]. Future studies should elucidate the underlying mechanisms by which the identified compounds affect LDs and reveal the cellular and physiological consequence of increasing LD size while simultaneously reducing their total quantity, before offering these compounds as a potential treatment option for CDS patients. Importantly, the selected compounds need to be further validated by orthogonal assays and their toxicity should be tested and characterized in the context of CDS patient cells. It is also important to check the potential off-target effects of the selected compounds. Yet, the data obtained from the conducted drug repurposing screen could set the basis for future CDS treatment avenues, and additionally, can be used to manipulate LDs for research purposes.

## Conclusions

By using primary skin fibroblasts derived from CDS patient, we revealed alternations in LDs, mitochondria and peroxisomes compared to control cells. Furthermore, we identified several compounds that affect LDs in the CDS patient cells and demonstrate that overexpressing functional ABHD5 diminishes LDs dramatically. Altogether, our work provides new insights into the molecular mechanism of CDS and suggests new avenues for developing treatments for CDS patients.

## Electronic supplementary material

Below is the link to the electronic supplementary material.

Supplementary Material 1

Supplementary Material 2

Supplementary Material 3

## Data Availability

All data supporting the findings of this study are available within the paper and its supplementary information. The results of the drug repurposing screen are provided in Supplementary Table 1. The Z factors of the screen are provided in Supplementary Table 2.
