# Supplementary material for: Using chanarin-dorfman syndrome patient fibroblasts to explore disease mechanisms and new treatment avenues
Source: Orphanet J Rare Dis. 2025 Apr 24;20:195. doi: 10.1186/s13023-025-03711-6 (PMC12020101; doi:10.1186/s13023-025-03711-6)
Supplement: Supplementary file 1 — Supplementary Material 1 [file 13023_2025_3711_MOESM1_ESM.docx]

**Supplementary Data**

**
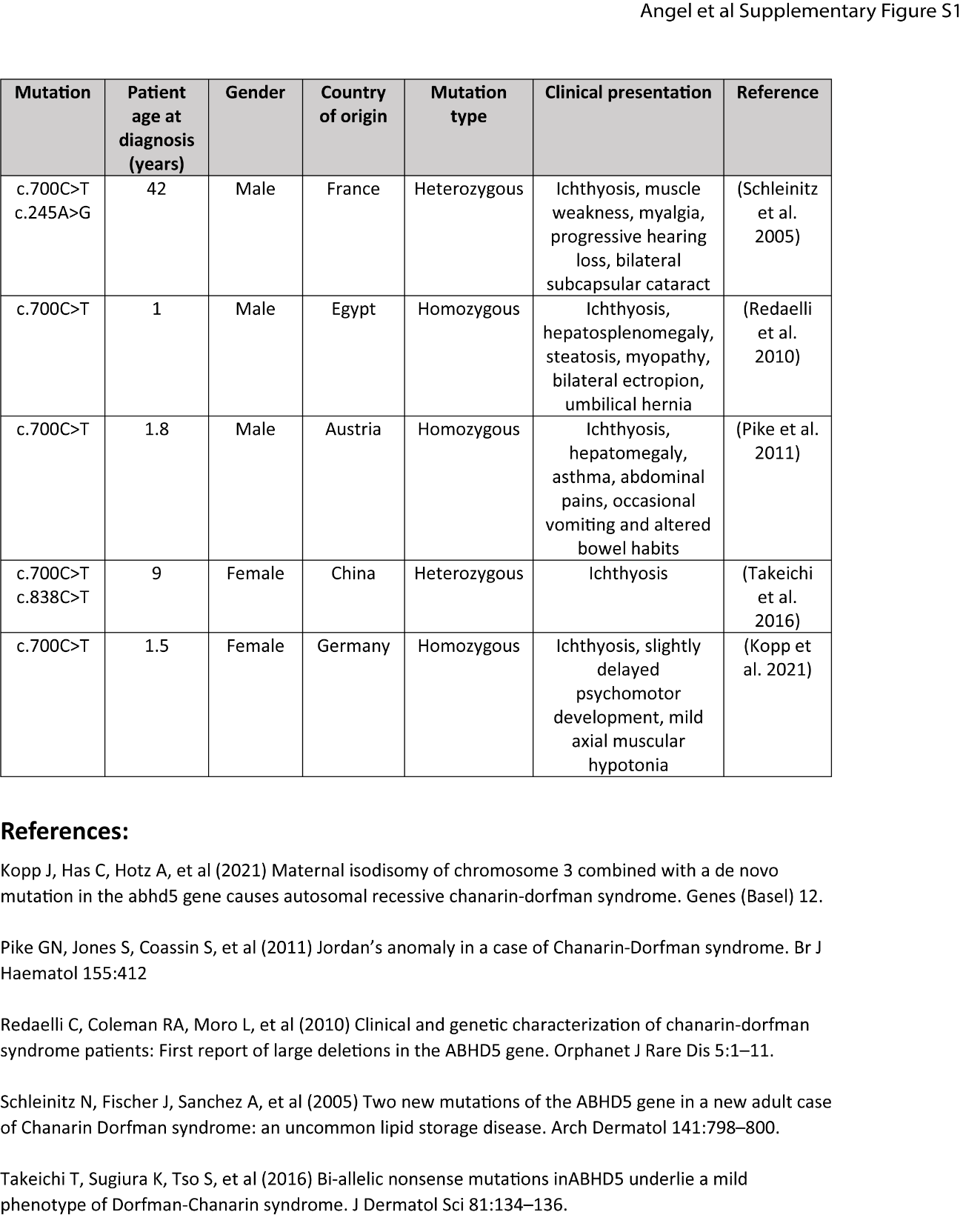
**

**Supplementary Figure S1:** A table summarizing all diagnosed c.700C>T mutations in CDS patients.


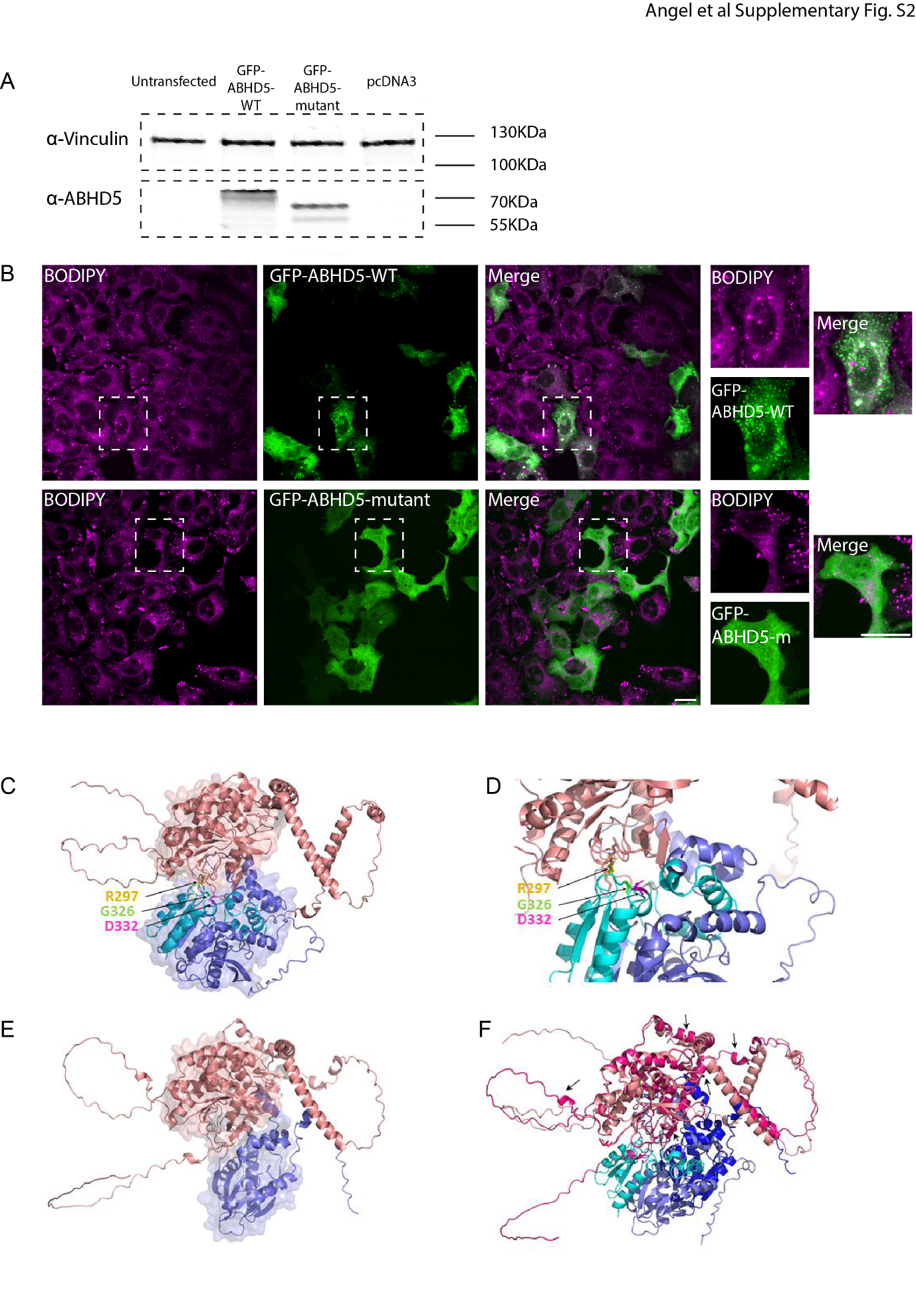


**Supplementary Figure S2: Truncated ABHD5 expressed in HeLa S3 cells does not localize to LDs.** (A) Western blot analysis of proteins extracts from HeLa S3 cell transfected with GFP-ABHD5-WT, GFP-ABHD5-mutant and pcDNA3 as a control (1 µg/ml, 24 h). Blots were incubated with anti-ABHD5 antibody recognizing the N’ of the protein and with anti-Vinculin as a loading control. (B) HeLa S3 cells were transfected for 24 h with GFP-ABHD5-WT or GFP-ABHD5-mutant (green). LDs were labeled with BODIPY (magenta) after fixation. Scale bars=20µm. Enlargements of designated areas are shown in the boxed regions at right. (C-E) AlphaFold model of the human ATGL/ABHD5 protein complex implies that the truncation of the C’ of ABHD5 affects its interaction with ATL.; (C) Human ATGL and human ABHD5 complex prediction by AlphaFold-Multimer, visualized in PyMOL in cartoon representation. The predicted structure of ATGL is in pink. The predicted structure of ABHD5 is in purple, with region truncated in the mutant (R234-D349) highlighted in cyan. High confidence predicted regions in both proteins are enclosed by the transparent pink and purple surfaces for ATGL and ABHD5 respectively. Three residues important for ATGL activation are colored in stick representation; R297 in yellow, G326 in green, and D332 in pink. (D) Close up of a groove in the human ATGL and human ABHD5 complex prediction by AlphaFold-Multimer where critical ABHD5 residues R297 (yellow), G326 (green) and D332 (pink) reside and are shown in stick representation. (E) Human ATGL and truncated human ABHD5 lacking 116 amino acids from the carboxyl-end (R234-D349) complex prediction by AlphaFold-Multimer, visualized in PyMOL in cartoon representation. The predicted structure of ATGL is in pink. The predicted structure of truncated ABHD5 is in purple. High confidence predicted regions in both proteins are enclosed by the transparent pink and purple surfaces for ATGL and ABHD5 respectively. (F) Superimposition of the predicted human ATGL and human ABHD5 complex with the predicted human ATGL and truncated human ABHD5 complex. Structures were predicted with AlphaFold-Multimer and superimposed with UCSF Chimera Matchmaker function. Arrows point to changes in ATGL conformation.


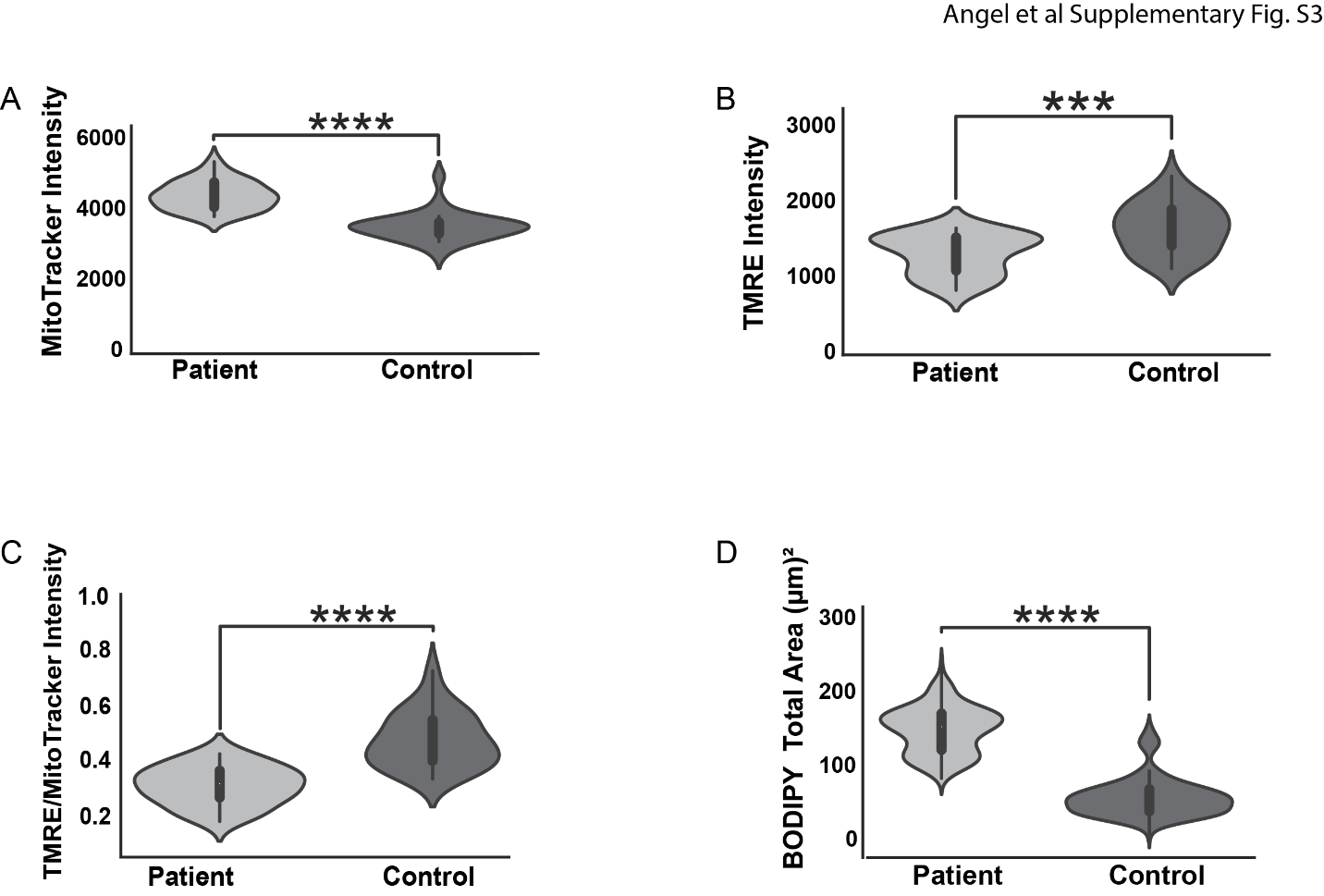


**Supplementary Figure S3: LDs and mitochondria are affected in patient fibroblasts compared to control skin fibroblasts (GM05400).** (A-C) Violin plots representing the intensity of MitoTracker (A), TMRE (B) or the ratio of TMRE\MitoTracker (C). Data were analyzed using two-sided T-test or two-sided Mann Whitney test. *****p*<0.0001, ****p*<0.001 n=1500 cells). (D) Violin plot representing total LD area as detected by BODIPY staining intensity. Data were analyzed by Mann-Whitney two-sided test, (*****p*<0.0001, n=1500 cells).

**Supplementary Table 1: Results of the drug repurposing screen.** A table summarizing the results of the drug repurposing screen analyzed by the IN CARTA 2200 image analysis software. The table includes: Compound name, Compound SMILES (Simplified Molecular Input Line Entry System of the compound), INCHIKEY (a textual identifier for the chemical substances), Compound ID (The Broad Institute ID of the compound), Compactness (SSMD (strictly standardized mean difference) score of the compactness of the organelles- average radius of the object calculated by the formula: (2 X Pi X gyration radius^2)/area), Form Factor (SSMD score of the form factor of the organelles. Form factor is the object roundness index with value from 0 to 1, where 1 is a circle), Area (SSMD score of the area of the organelles), Total Area (SSMD score of the total area of the organelles. Total area is the sum of area of all the organelles), Survival (the survival of the cells with a given compound, calculated by the number of nuclei in a well / number of nuclei in negative control (DMSO), Final status (if the compound was considered as hit or not).

**Supplementary Table 2: Z’-factors calculated for the drug repurposing screen.**
